# Regulation of cardiac fibroblast cell death by unfolded protein response signaling

**DOI:** 10.3389/fphys.2023.1304669

**Published:** 2024-01-12

**Authors:** Mary B. Rowland, Patrick E. Moore, Robert N. Correll

**Affiliations:** ^1^ Department of Biological Sciences, University of Alabama, Tuscaloosa, AL, United States; ^2^ Center for Convergent Bioscience and Medicine, University of Alabama, Tuscaloosa, AL, United States

**Keywords:** unfolded protein response, er stress, cardiac fibroblast, fibrosis, cell death, apoptosis

## Abstract

The endoplasmic reticulum (ER) is a tightly regulated organelle that requires specific environmental properties to efficiently carry out its function as a major site of protein synthesis and folding. Embedded in the ER membrane, ER stress sensors inositol-requiring enzyme 1 (IRE1), protein kinase R (PKR)-like endoplasmic reticulum kinase (PERK), and activating transcription factor 6 (ATF6) serve as a sensitive quality control system collectively known as the unfolded protein response (UPR). In response to an accumulation of misfolded proteins, the UPR signals for protective mechanisms to cope with the cellular stress. Under prolonged unstable conditions and an inability to regain homeostasis, the UPR can shift from its original adaptive response to mechanisms leading to UPR-induced apoptosis. These UPR signaling pathways have been implicated as an important feature in the development of cardiac fibrosis, but identifying effective treatments has been difficult. Therefore, the apoptotic mechanisms of UPR signaling in cardiac fibroblasts (CFs) are important to our understanding of chronic fibrosis in the heart. Here, we summarize the maladaptive side of the UPR, activated downstream pathways associated with cell death, and agents that have been used to modify UPR-induced apoptosis in CFs.

## 1 Introduction

**FIGURE 1:**
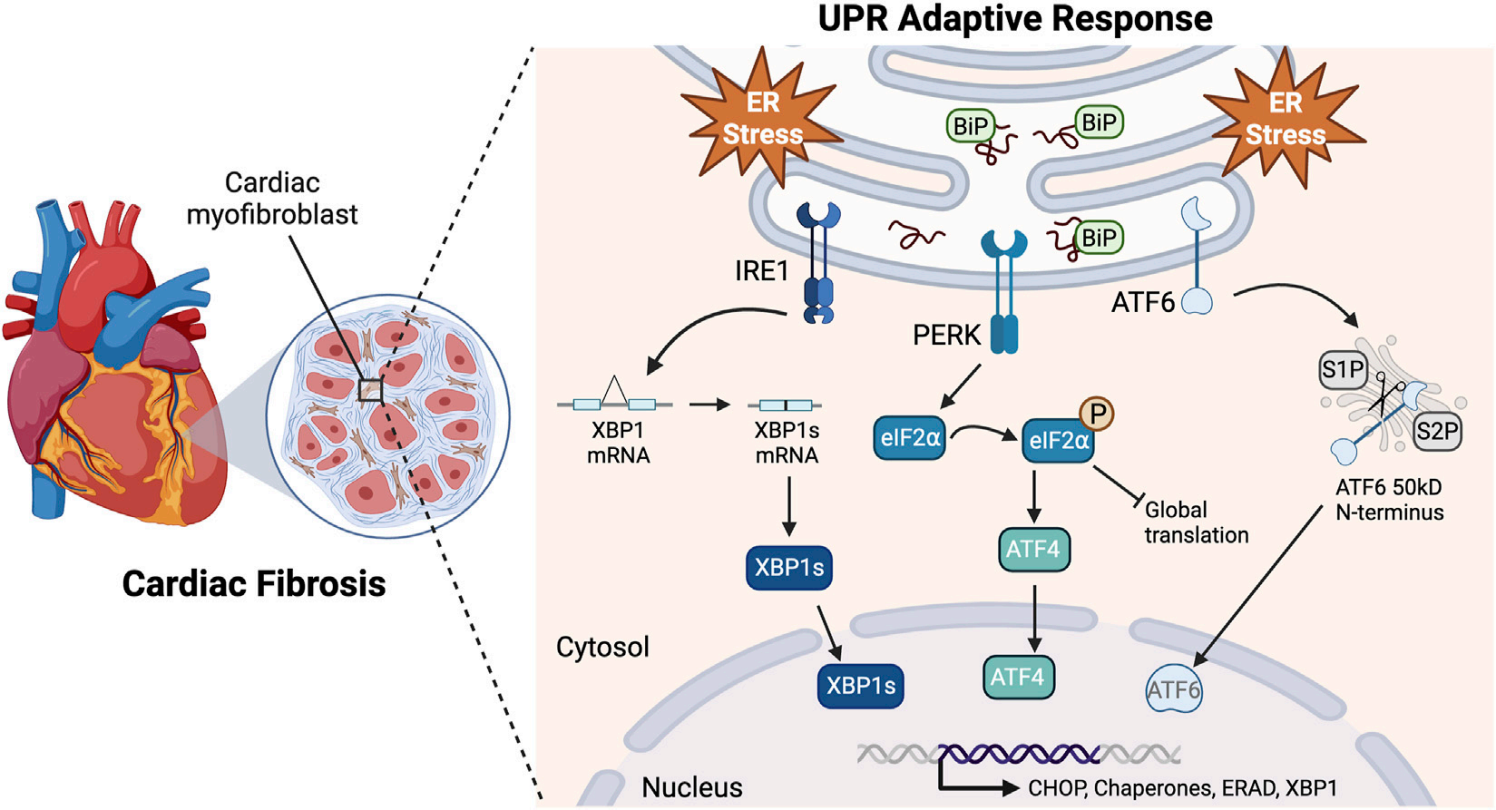
Adaptive UPR signaling pathways. Upon accumulation of misfolded proteins and ER stress, BiP (GRP78) disassociates from the UPR sensors to act as a chaperone. This allows IRE1 homodimerization causing the RNase domain to splice XBP1 mRNA resulting in the expression of XBP1s. PERK also homodimerizes, resulting in kinase activity that phosphorylates eIF2⍺. This leads to a global translation block and a reading frame shift in ATF4 that allows it to escape the block. ATF6 translocates to the Golgi apparatus where it is cleaved by proteases S1P and S2P, releasing its 50kD N-terminus. These pathways lead to the translocation of transcriptionally active XBP1s, ATF4, and N-ATF6 to the nucleus to upregulate UPR genes in response to ER stress Created with BioRender.com.

Understanding the signaling cascades responsible for CF apoptosis could uncover ways to ameliorate chronic cardiac fibrosis (Matsumoto et al., 1996; Han et al., 2009; Groenendyk et al., 2016; Ren et al., 2021). Fibroblasts secrete and maintain tissue extracellular matrix (ECM) and, when differentiated into myofibroblasts, they enhance the matrix to aid in cell migration, communication, and wound healing (Galbraith and Sheetz, 1998; Brown et al., 2007; Jellis et al., 2010; van Nieuwenhoven and Turner, 2013). ER stress results from many cardiac pathologies, and homeostasis is maintained by signaling through the three arms of the UPR: IRE1, PERK, and ATF6 (Figure 1) (Minamino and Kitakaze, 2010; Arrieta et al., 2018). While the UPR is typically protective, under prolonged stressful conditions, such as oxidative stress, proteotoxicity, or impaired calcium signaling, it may shift to maladaptive signaling resulting in programmed cell death (Liang et al., 2012; Muchowicz et al., 2015; Losada et al., 2020).

UPR-induced apoptosis may be divided into three phases that include initiation, commitment, and execution (Szegezdi et al., 2006a). Unresolvable stress initiates signaling, causing a commitment to apoptosis by upregulation of CCAAT enhancer-binding protein C/EBP homologous protein (CHOP) by all three arms, and an execution phase through downstream caspase activation (Matsumoto et al., 1996; Zinszner et al., 1998; Szegezdi et al., 2006b; Yang et al., 2020). The mechanisms that contribute to each phase have not been well characterized in CFs. Most studies have been performed *in vitro*, leaving many unanswered questions, such as how UPR signaling contributes to *in vivo* replacement, interstitial, and perivascular fibrosis (Factor et al., 1991; Aoki et al., 2011; Dai et al., 2012). Here, we review the known maladaptive pathways of the UPR, the current literature available about the role of the UPR in CF apoptosis, and areas of expansion needed in this field.

## 2 The UPR in cardiac fibroblast cell death

Maladaptive downstream effectors of the UPR, such as Jun-N-terminal kinase (JNK) and the B-cell lymphoma-2 (Bcl-2)/Bax ratio, have been assessed as a standard indicator for cell death in CFs (Tian et al., 2002; Zhao and Eghbali-Webb, 2002; Mayorga et al., 2004; Lai et al., 2009; Ghavami et al., 2012a; Ghavami et al., 2012b; Parra-Flores et al., 2021). Ghavami, et al. investigated maladaptive UPR signaling in the context of the mevalonate cascade, a pathway important in cholesterol synthesis (Ghavami et al., 2012a). Clinically, statins, which inhibit 3-hydroxy-3-methyl-glutaryl-CoA reductase (HMG-CoA), have been observed to decrease cardiac remodeling and activate apoptosis (Ghavami et al., 2012a). It was reported that inhibiting HMG-CoA with simvastatin simultaneously activated apoptosis and the UPR in human atrial fibroblasts and could be reversed by exposure to exogenous mevalonate (Ghavami et al., 2012a). This was supported with evidence that IRE1, cleaved ATF6, phosphorylated PERK, and CHOP expression increased upon simvastatin treatment. Spliced X-box binding protein 1 (XBP1) had the most significant increase in expression compared to all the UPR components examined (Ghavami et al., 2012a). This was further substantiated by characterization of maladaptive downstream molecules of IRE1, such as JNK, p53-upregulated modulator of apoptosis (PUMA), NOXA, and Bcl-2/Bax (Ghavami et al., 2012a). There was a significant increase in Bax, PUMA, NOXA, and caspase-3/7/9 expression with a simultaneous decrease in Bcl-2 and Mcl-1 (Ghavami et al., 2012a). The decrease in Bcl-2 was reversed by JNK inhibition, indicating that JNK signaling may be responsible for additional promotion of apoptotic effects which the authors suggest is limited by autophagic flux (Ghavami et al., 2012a). By performing a 3-(4,5dimethylthiazol-2-yl)-2,5-diphenyl-2H-tetrazolium bromide (MTT) assay for cell viability, the data suggested that an increase in IRE1 expression was accompanied with an increase in human atrial fibroblast death (Ghavami et al., 2012a). The authors did not investigate the direct relationship between ATF6 activation and CHOP-mediated apoptosis, leaving it undetermined if ATF6 signaling orchestrated the observed maladaptive cell death pathways. Further clarifying the association between CF apoptosis and the Bcl-2/Bax ratio, Ghavami et al. exposed rat ventricular myofibroblasts to the trans-fatty acids (TFAs), vaccenic acid (VA) and elaidic acid (EA), at concentrations of 200 and 400 µM (Ghavami et al., 2012b). These treatments resulted in a significant increase in the percentage of apoptosis and a significant decrease in cell viability (Ghavami et al., 2012b). This was corroborated with a significant decrease in Bcl-2/Bax ratio (Ghavami et al., 2012b). Although TFAs are known ER stressors and UPR inducers, they are reported to not all have the same effect on cellular homeostasis and additional work is required to establish that specific TFAs (such as VA and EA) induce CF apoptosis through maladaptive UPR mechanisms (Oteng and Kersten, 2020).

Most of the work investigating the UPR in CF cell death has been completed *in vitro*, so more *in vivo* experiments are necessary to understand the broader physiological impacts of these pathways. This is particularly relevant when investigating CFs because they are mechanosensitive while functioning in an environment with repeated movement and a precise matrix composition and stiffness. In a recent study by Parra-Flores and collaborators, an *in vivo* model of ischemia/reperfusion (I/R), a known activator of the UPR, was used (Zhang et al., 2017; Parra-Flores et al., 2021). They saw a decrease in neonatal CF viability and an increase in apoptotic signaling following I/R; these effects were recovered by antioxidant exposure (Parra-Flores et al., 2021). This was associated with toll-like receptor 4 activation, a known regulator of IRE1 and XBP1 (Martinon et al., 2010; Parra-Flores et al., 2021). Further analyses showed decreases in pro-caspase 3/9 expression, decreases in p38 MAPK and JNK phosphorylation, and an increase in the Bcl-2/Bax ratio (Parra-Flores et al., 2021). Because all these components are important downstream players in maladaptive IRE1 signaling, expanding this work could clarify how the UPR may orchestrate pathways leading to CF apoptosis (Wang and Ron, 1996; Griffiths et al., 2001; Zong et al., 2001; Hitomi et al., 2004; Kato et al., 2012; Parra-Flores et al., 2021).

## 3 IRE1 in cell death

Irreversible ER stress causes IRE1 dimerization, resulting in recruitment of tumor necrosis factor receptor associated factor 2 (TRAF2) and apoptotic-signaling-kinase 1 (ASK1) that leads to maladaptive signaling (Urano et al., 2000; Nishitoh et al., 2002; Luo et al., 2008). The most referenced maladaptive downstream player of IRE1, JNK, activates apoptotic pathways and phosphorylates the anti-apoptotic family of Bcl-2 proteins (Lei and Davis, 2003; Wei et al., 2008; Shimizu et al., 2010; Kato et al., 2012). The upregulation of CHOP by the IRE1-TRAF2-ASK1 complex increases apoptotic proteins, such as Bcl-2-like 11 (BIM) and death receptor 5 (DR5), while simultaneously suppressing anti-apoptotic gene expression such as Bcl-2 (Wang and Ron, 1996; McCullough et al., 2001; Ghosh et al., 2012; Jung et al., 2015). The IRE1-TRAF2-ASK1 complex also activates caspases, such as caspase-12 and caspase-3, required for apoptosis (Yoneda et al., 2001; Hitomi et al., 2004).

### 3.1 IRE1 in cardiac fibroblast cell death

The significance of maladaptive downstream signalers of IRE1 in CF apoptosis can be supported through the work done by Feng and collaborators (Feng et al., 2018). These authors showed that the elevated levels of BiP, CHOP, PUMA, and caspase-3 protein resulting from transverse aortic constriction (TAC) could be significantly reduced when these mice were treated with hydrogen sulfide (H_2_S) (Feng et al., 2018). It was unclear if these expression levels were specifically due to CFs or other cardiac cell types, but these effects were supported *in vitro* through H_2_S attenuation of hydrogen peroxide (H_2_O_2_)-mediated apoptosis in isolated human CFs (Feng et al., 2018).

The Bcl-2/Bax ratio is regulated downstream of IRE1 and influences CF apoptosis (Mayorga et al., 2004; Shemorry et al., 2019). siRNA knockdown of Bcl-2 resulted in an increase in CF apoptosis (Mayorga et al., 2004). JNK phosphorylation can inhibit Bcl-2 function and reduce the cell’s ability to properly regulate Ca^2+^ homeostasis in the ER and increase mitochondrial Ca^2+^ uptake (Murphy et al., 1996; He et al., 1997; Lei and Davis, 2003; Scorrano et al., 2003; Wei et al., 2008). Indeed, IRE1 can also activate JNK through TRAF2 and ASK1 leading to disinhibition of Bax/Bak by Bcl-2, and enabling cytochrome c release from the mitochondria (Gorman et al., 2012). Mitochondrial-mediated apoptosis was associated with shifts in Bcl-2, Bax, or caspase expression (Tian et al., 2002). Tian et al. treated rat CFs with inflammatory cytokines, which induced nitric oxide-mediated apoptosis (Tian et al., 2002). This exposure was reported alongside a significant 3.5-fold increase in Bax, a 2.5-fold increase in caspase-3 expression, and a 7-fold increase in caspase-3 activity (Tian et al., 2002). Meanwhile, Lai and others showed that higher doses of norepinephrine up to 100 µM significantly increased apoptosis and decreased the number of viable rat CFs (Lai et al., 2009). This exposure was reported to be associated with an increase in Bax mRNA expression and caspase-3 activity, indicating cytotoxicity and the activation of apoptotic pathways (Lai et al., 2009). Although these authors did not identify the upstream mediators of these results, it has been demonstrated that norepinephrine can induce UPR signaling, specifically ATF6 and IRE1, in HepG2 cells, human fat explants, and 3T3-L1 mouse adipocytes (Lai et al., 2009; Abdullahi et al., 2020).

Interestingly, Zhao and collaborators looked at differences by sex of rat CFs to apoptotic stimuli (Zhao and Eghbali-Webb, 2002). They found that following 15 min of hypoxia, isolated CFs from males had a steeper increase in JNK expression relative to females in comparison to each of their basal levels, but females had an overall higher basal JNK expression compared to male CFs (Zhao and Eghbali-Webb, 2002). Evidence in rodents and humans supports that there is less cardiac remodeling and fibrosis in females (Kessler et al., 2019). Because sex differences are associated with human cardiovascular disease progression and outcomes, this perspective of CF cell death is worth exploring further. Of particular significance when considering CFs *in vitro* is the absence of intrinsic, sex-based hormonal signaling, such as estrogen, a known contributor to enhanced wound repair. With this, the lack of complex sex specific characteristics in cell culture should be taken into consideration. Other tissue types, such as the kidney, have also shown sex differences in UPR signaling through increased ER stress markers and apoptosis in tunicamycin-treated male mice, compared to females (Hodeify et al., 2013). Using the perspective taken by Zhao, et al., the potential role of UPR signaling through JNK and its apoptotic effects in CFs could further clarify factors contributing to the observed sex differences in cardiac outcomes.

## 4 PERK in cell death

The serine/threonine kinase activity of PERK phosphorylates eukaryotic translation initiation factor 2a (eIF2⍺) (Koumenis et al., 2002; Cui et al., 2011). This attenuates overall translation while increasing the translation of specific mRNAs such as activating transcription factor 4 (ATF4), critical for the transcription of CHOP (Fawcett et al., 1999; Blais et al., 2004). CHOP upregulates three important components of UPR-induced apoptosis which are *tribbles*-related protein 3 (TRB3), DR5, and growth arrest and DNA damage-inducible gene 34 (GADD34) (Marciniak et al., 2004; Yamaguchi and Wang, 2004; Ohoka et al., 2005). TRB3 prevents proliferation, transcription, and differentiation signaling by binding Akt and inhibiting phosphorylation (Du et al., 2003). Apoptotic signaling is enhanced through DR5 by the activation of caspases (Tanner and Grisanti, 2021). When ER stress cannot be reversed, GADD34 binds protein phosphatase-1⍺ to dephosphorylate eIF2⍺ and remove the translation block which is associated with apoptosis (Brush et al., 2003; Choy et al., 2015; Collier et al., 2015). Pro-apoptotic signaling through CHOP also influences Bax/Bak and outer mitochondrial membrane permeabilization by reducing Bcl-2 and increasing BIM (Puthalakath et al., 2007; Luna-Vargas and Chipuk, 2016; Zhou et al., 2019).

### 4.1 PERK in cardiac fibroblast cell death

PERK may orchestrate maladaptive effects in CFs because multiple downstream PERK signaling molecules, such as CHOP, ATF4, DR5, and Bcl-2, have been shown to influence CF apoptosis (Mayorga et al., 2004; Humeres et al., 2014; Sokolova et al., 2017; Feng et al., 2018; Olivares-Silva et al., 2021; Tanner and Grisanti, 2021). Since all three arms of the UPR regulate CHOP signaling, defining which branch has the most impact on CF apoptosis is critical for fully explaining the role of CHOP in this process (Yang et al., 2020). Recently, Olivares-Silva and others reported that ER stress induced by tunicamycin, ischemia, and I/R increased CHOP protein expression in neonatal Sprague Dawley rat CFs (Olivares-Silva et al., 2021). This was associated with an increase in apoptosis and reduction of viability in a time and concentration-dependent manner (Olivares-Silva et al., 2021). Similarly, Humeres and collaborators isolated CFs from neonatal Sprague-Dawley rats and induced ER stress through thapsigargin treatments (Humeres et al., 2014). This increased GRP78, protein disulfide-isomerase (PDI), ATF4, and CHOP protein levels while simultaneously decreasing cell viability in a time and concentration-dependent manner (Humeres et al., 2014). Work by Feng et al. found that exposure to H_2_O_2_ resulted in a significant decrease in human CF viability and an increase in apoptosis in a dosage dependent manner, which occurred concurrently with an increase in CHOP expression and could be ameliorated by exposure to H_2_S (Feng et al., 2018). Together, these investigations provide evidence that downstream mediators of PERK signaling, such as CHOP and ATF4, modulate CF cell viability.

Sokolova and coauthors recently found that palmitate (PA), a saturated fatty acid found in plasma, induced ER stress in adult mouse CFs (Sokolova et al., 2017). PA increased the gene expression of CHOP and ATF4 while also increasing CF apoptosis and decreasing CF contractile function (Sokolova et al., 2017). Annexin V-fluorescein isothiocyanate (FITC) labeling detected a significant increase in early-stage apoptosis while propidium iodide binding to nuclear DNA did not show a significant late-stage apoptosis/necrosis when treated with PA (Sokolova et al., 2017). This is particularly interesting because it has been reported that PERK induction of CHOP is more significant in the later stages of apoptosis, as indicated by propidium iodide staining, in other cell types (Lu et al., 2017; Liu and Zhang, 2020). It may be useful to apply these apoptotic stage analyses to evaluate the timing of maladaptive signaling within each of the UPR branches. Further, determining the time point of each switch between adaptive and maladaptive UPR signaling could have clinical applications in treatments targeting these mechanisms.

PERK is a known upstream mediator of DR5 (Lu et al., 2014). Tanner and others showed that DR5 signaling was correlated with proliferation in inactivated ventricular fibroblasts but apoptosis in activated ventricular myofibroblasts (Tanner and Grisanti, 2021). This was evident through a significant increase in DR5 expression, caspase 3/7 activity, and apoptosis, measured by terminal deoxynucleotidyl transferase dUTP nick end labeling (TUNEL) staining, in myofibroblasts compared to fibroblasts (Tanner and Grisanti, 2021). This was further substantiated *in vivo* by Masson’s Trichome staining, which showed that after isoproterenol injection there was an increase in fibrosis in DR5 gene-deleted mice compared to control mice (Tanner and Grisanti, 2021). Studies such as this that compare UPR signaling in fibroblasts and myofibroblasts could help to clarify the roles of these signaling pathways in CF cell death.

## 5 ATF6 in cell death

Elevated ATF6 expression, due to either disease processes or viral transduction, is associated with increased cellular apoptosis (Morishima et al., 2011; Tan et al., 2020). In colorectal cancer cells, the ATF6 transcriptionally active N-terminus increased GRP78*,* DDIT3 (which encodes CHOP)*,* and EIF2AK3 (which encodes PERK) gene expression and significantly increased apoptotic cells (Spaan et al., 2019). Similarly, overexpression of ATF6 increased CHOP and Bax mRNA and protein levels, decreased Bcl-2 expression, and significantly increased the rate of apoptosis (Huang et al., 2018). Depletion of ATF6 decreased CHOP expression and increased Bcl-2 expression, resulting in a decrease of apoptosis (Xiong et al., 2017). Upon ATF6 silencing, pro-apoptotic effects of hydroxycamptothecin on fibroblasts was significantly weakened (Wei et al., 2018; Yao et al., 2019; Tao et al., 2021). Further examination of the PERK/p-eLF2α/ATF4 pathway could elucidate ATF6’s role in apoptosis, as it has been reported that eIF2⍺ phosphorylation and ATF4 activation are necessary for ATF6 activation (Teske et al., 2011).

### 5.1 ATF6 in cardiac fibroblast cell death

Little work has been done to examine the role of ATF6 signaling in CF apoptosis. ATF6 activity is generally protective in the heart, but it is unclear how its signaling affects CF activity and survival (Toko et al., 2010; Glembotski et al., 2019). Data presented by Toko and collaborators showed that inhibition of ATF6 with 4-(2-aminoethyl) benzenesulfonyl fluoride or knockdown of ATF6 with siRNA decreased cardiac function, increased myocardial infarction mortality rate, and increased cardiomyocyte apoptosis in mice (Toko et al., 2010). However, ATF6 is also known to transcriptionally upregulate the important UPR-mediated proapoptotic molecule, CHOP (Yoshida et al., 2000; Yang et al., 2020).

The role of ATF6 in CF function has recently been expanded on in Stauffer, et al. (Stauffer et al., 2020). It was reported that pharmacologic activation of ATF6 using compound 147 in murine ventricular fibroblasts resulted in a decrease in fibroblast activation and contraction while the opposite was seen in siRNA knockdown of ATF6 (Stauffer et al., 2020). However, ATF6 effects on apoptosis and cell viability were not measured (Stauffer et al., 2020). Because CHOP upregulation is not entirely dependent on ATF6 signaling, many experiments did not consider the potential contribution of the ATF6 pathway in their analyses. Because ATF6 has not been fully explored and its significance to maladaptive UPR signaling in CFs is unclear, it is a plausible target for further investigation.

## 6 Cardiac fibroblast resistance to apoptosis

Fibroblasts have unique characteristics and gene expression patterns that are organ-specific (Lindner et al., 2012). In most tissues, fibroblasts undergo apoptosis following scar formation (Desmouliere et al., 1995). However, in the heart, activated and ⍺SMA-expressing cardiac myofibroblasts have been found in the infarct scar up to 17 years following an initial cardiac event (Willems et al., 1994). Continuous presence of myofibroblasts results in excessive synthesis and secretion of ECM components causing ventricular stiffness and heart failure (van den Borne et al., 2010). Elucidating the specific regulatory mechanisms that allow CFs to elude apoptosis more frequently than other tissue fibroblasts could identify key features of how the UPR may contribute to chronic cardiac fibrosis.

A possible explanation for CF apoptosis evasion is through distinctive extrinsic pro-survival conditions, such as integrin-mediated transduction, paracrine factors, or a specific composition of ECM network resulting from the electrical and mechanical stimuli in the heart (Huebener et al., 2008; Cai et al., 2019; Titus et al., 2021). Another explanation for enhanced apoptotic resistance is that the augmented pro-survival pathways are more magnified than the maladaptive signals, causing an increase in fibroblast viability (Bea et al., 2022). CFs could also have heightened resistance to apoptosis through response to intrinsic signaling and continual autophagy (Zeglinski et al., 2016). It has been shown that activated cardiac myofibroblasts are more resistant to apoptosis than quiescent CFs, indicating enhanced pro-survival molecular mechanisms in those cells (Lagares et al., 2017; Hinz and Lagares, 2020). Some of the suggested cytoprotective molecular mechanisms aiding in apoptosis avoidance include canonical Transforming Growth Factor β (TGFβ) signaling, a decrease in Bax and caspase expression, and an increase in Bcl-2 expression (Mayorga et al., 2004; Anuka et al., 2013; Vivar et al., 2013; Olivares-Silva et al., 2021). The UPR is a potential candidate to investigate CF survival due to its crosstalk with TGFβ signaling and its response to Bcl-2 upregulation (Mayorga et al., 2004; Vivar et al., 2013; Olivares-Silva et al., 2021).

## 7 Discussion

ER stress and the UPR are contributors to various cardiac pathologies such as hypertrophy, ventricular dysfunction, and heart failure (Park et al., 2012). There is a significant amount of literature describing the relevance of the UPR in apoptosis in other cell types or in fibroblasts of other tissues (Shi et al., 2013; Hong et al., 2015; Tang et al., 2016; Delbrel et al., 2018; Pibiri et al., 2020). UPR regulation of CF cell death is relatively understudied in the context of reducing pathological cardiac fibrosis. Downstream effectors of IRE1 such as Bax, Bcl-2, PUMA, JNK, and caspase-3 have been reported to be involved in CF apoptosis (Tian et al., 2002; Mayorga et al., 2004; Lai et al., 2009; Ghavami et al., 2012a; Ghavami et al., 2012b; Feng et al., 2018; Parra-Flores et al., 2021). Research has also affirmed that activation of ATF6 upregulates CHOP, but the extent to which this regulates CF apoptosis has not been explored (Yoshida et al., 2000; Ghavami et al., 2012a; Yang et al., 2020). All three UPR arms upregulate CHOP, but the PERK pathway is essential for CHOP expression in comparison to IRE1 and ATF6 (Humeres et al., 2014; Sokolova et al., 2017; Olivares-Silva et al., 2021). Therefore, this may suggest that PERK-ATF4-CHOP signaling is the most influential UPR arm in CF apoptosis.

Much of the work reviewed did not explicitly identify the specific UPR arm influencing CF cell death and instead looked at their downstream mediators. Expanding these investigations can determine if these downstream molecules are signaled to through a specific UPR pathway or an alternative upstream mechanism. Additionally, studies investigating UPR-mediated CF apoptosis use a variety of methods to induce cellular stress that may result in different ER stress mechanisms being activated and variations in severity that could obfuscate our understanding. Future work exploring these mechanisms will provide a better understanding of chronic fibrosis, CF apoptotic resistance, and potential pharmacological manipulations that might provide new therapies for various cardiovascular pathologies.
